# DIC Score Combined With CLIF-C OF Score Is More Effective in Predicting Prognosis in Patients With Hepatitis B Virus Acute-on-Chronic Liver Failure

**DOI:** 10.3389/fmed.2022.815580

**Published:** 2022-02-11

**Authors:** Xueyun Zhang, Jiajia Han, Xun Qi, Yao Zhang, Pu Zhou, Xiaoqin Liu, Yue Ying, Wenhong Zhang, Jiming Zhang, Yuxian Huang

**Affiliations:** ^1^Department of Infectious Diseases, Shanghai Key Laboratory of Infectious Diseases and Biosafety Emergency Response, National Medical Center for Infectious Diseases, Huashan Hospital, Fudan University, Shanghai, China; ^2^Department of Hepatology, Shanghai Public Health Clinical Center, Fudan University, Shanghai, China; ^3^Huashan Worldwide Medical Center, Huashan Hospital, Fudan University, Shanghai, China; ^4^Key Laboratory of Medical Molecular Virology (MOE/MOH), Shanghai Institute of Infectious Diseases and Biosecurity, Shanghai Medical College, Fudan University, Shanghai, China; ^5^Department of Infectious Diseases, Jing'an Branch of Huashan Hospital, Fudan University, Shanghai, China

**Keywords:** prognosis, acute-on-chronic liver failure, DIC score, coagulation, prognostic score

## Abstract

Coagulation and fibrinolysis disorders are major prognostic factors in hepatitis B virus-related acute-on-chronic liver failure (HBV-ACLF) patients. Here, we aimed to clarify the role of disseminated intravascular coagulation (DIC) scores in predicting HBV-ACLF patient prognosis. We assessed the DIC score from HBV-ACLF patients at Huashan Hospital in Shanghai, China from June 2013 to May 2021 and evaluated it in relation to short-term mortality, clinical course, and infection. A novel prognostic scoring model was proposed based on DIC scores. A total of 163 transplant-free HBV-ACLF patients were enrolled. DIC scores were higher in non-survivors than survivors (6 vs. 4, *P* = 0.000) and were independently associated with short-term mortality [hazard ratio (HR): 1.397, 95% confidence interval (95% CI): 1.040–1.875, *P* = 0.026]. DIC scores were associated with ACLF grade, clinical course, and infection. Moreover, they were correlated with model for end-stage liver disease (MELD) scores (*r* = 0.521, *P* < 0.001). The area under the receiver operating curve (auROC) of CLIF-C OF-DICs [a novel prognostic score based on age, DIC score, and Chronic liver failure-consortium organ function score (CLIF-C OFs)] for 90-day mortality was 0.936, which was higher than six other generic prognostic scoring models. These results were confirmed in a validation cohort (*n* = 82). In conclusion, elevated DIC score is associated with poor prognosis in HBV-ACLF patients, and can be used jointly with CLIF-C OFs to improve the accuracy of prognosis prediction.

## Introduction

Acute-on-chronic liver failure (ACLF) is a syndrome characterized by acute deterioration of pre-existing chronic liver disease and associated with substantial short-term mortality (1), with an overall 28-day mortality of 30–50% and a 90-day mortality of 50–80%. In the Asian-Pacific and African regions, hepatitis B virus (HBV) infection is the main cause of ACLF, resulting in HBV-related ACLF (HBV-ACLF). Its common precipitating factor is hepatitis B flare (2), and it presents with higher severity and mortality as well as a higher prevalence of liver and coagulation failure.

Coagulation disorders are common in ACLF, especially in HBV-ACLF, and they play a significant role in the prognosis evaluation of liver disease (3). The coagulation system consists of a coagulation promoting system and an anticoagulation system. The former mainly involves platelets and clotting factors, while the latter includes anticoagulants and the fibrinolytic system (4). In ACLF, the patient's perturbed hemostatic system is often precariously rebalanced by off-setting factors, with declines in thrombocytes, liver-derived procoagulant factors (such as factors V, VII, and X), and anticoagulant factors (especially protein C) concurrent with increases in endothelial-derived von Willebrand factor (VWF) and factor VIII. The net result is a thrombin-generating capacity comparable to or even increased relative to healthy individuals (5, 6). The international normalized ratio (INR) is the most widely used indicator of ACLF prognosis (1, 2, 7–9). It mathematically standardizes prothrombin time (PT) to allow PT results from different laboratories to be compared. However, it is accurate only for values within 1.5–4.5, and it only reflects the extrinsic coagulation pathway and does not include liver-derived anticoagulant factors (4). The disseminated intravascular coagulation (DIC) score is a classical diagnostic scoring system; it includes platelet counts, fibrin-related markers, fibrinogen, and PT (10). Consequently, it more comprehensively reflects the coagulation system than any single standard coagulation test. DIC is a syndrome characterized by widespread intravascular activation of coagulation leading to substantial fibrin deposition. It can be induced by infection (such as sepsis) or non-infection (such as severe hepatic failure) (11). DIC score has been demonstrated to be an independent predictor of organ failure and mortality in sepsis (12). The effects of sepsis on coagulation are complex and are similar to those of ACLF on hemostasis, especially when sepsis-induced DIC occurred (6, 13). However, no study has yet explored the relationship between DIC score and ACLF.

The purpose of this retrospective, single-center study was to assess the association between DIC score and HBV-ACLF patient prognosis, as well as to build a novel prognostic scoring model based on DIC score that can help with treatment decisions for HBV-ACLF patients in the clinic.

## Methods

### Patients

**Figure 1:**
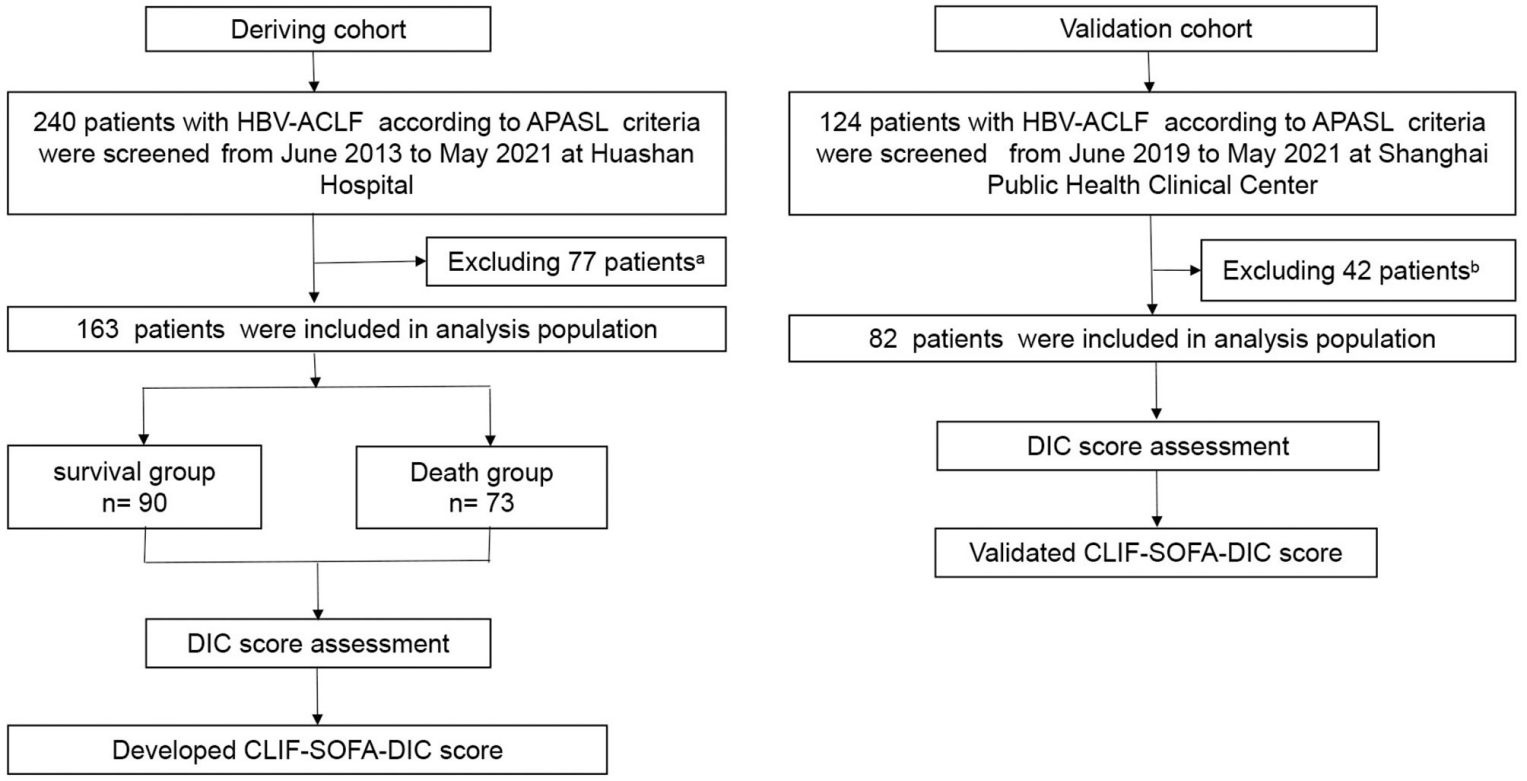
Flow chart of the study design. ^*a*^A total of 77 patients from the derivation cohort were excluded for one or more of the following reasons: 29 had hepatocellular carcinoma or other tumors; 12 had severe extra-hepatic diseases; 6 were receiving immunosuppressive drugs for reasons other than chronic liver diseases; 6 complicated with alcoholic hepatitis; 1 complicated with schistosomiasis cirrhosis; 2 complicated with Wilson disease; 14 had received liver transplants within 28 days; and 7 were lost to follow-up within 28 days. ^*b*^A total of 42 patients in validation cohort were excluded for one or more of the following reasons: 13 had hepatocellular carcinoma or other tumors; 8 had severe extra-hepatic diseases; 2 were receiving immunosuppressive drugs for reasons other than chronic liver diseases; 12 complicated with alcoholic hepatitis; 1 complicated with schistosomiasis cirrhosis; 3 patients received liver transplants within 28 days; and 3 were lost to follow-up within 28 days. HBV-ACLF, hepatitis B virus-related acute-on-chronic liver failure; APASL, Asian Pacific Association for the Study of the Liver.

Two cohorts were enrolled in this study. For the derivation cohort, 240 patients at Huashan Hospital, Fudan University, a tertiary hospital in Shanghai, China, from June 2013 to May 2021 who met the Asian Pacific Association for the Study for the Liver (APASL) HBV-ACLF criteria were consecutively enrolled; 77 were excluded. Sixteen patients withdrew during the third month of follow-up. The validation cohort included 82 patients treated at the Shanghai Public Health Clinical Center from May 2019 to May 2021 (detailed inclusion and exclusion criteria are described in Figure 1 and Supplementary Method 1). This study was conducted in accordance with the Helsinki Declaration and was approved by the Ethical Committee of Huashan Hospital of Fudan University and the Ethical Committee of the Shanghai Public Health Clinical Center. Written informed consent was obtained from each patient or their legal representative.

### Data Collection

Detailed clinical characteristics were collected from medical records or the hospital database, and these included blood parameters (coagulative function, routine blood tests, biochemical examination, alpha fetoprotein, and HBV index), history of chronic disease, potential precipitating events, history of antiviral therapy, ascites, infection, organ failure, and treatments received. Chinese Group on the Study of Severe Hepatitis B-ACLF (COSSH-ACLF) grades, European Association for the Study of the Liver-ACLF (EASL-ACLF) grades and DIC score were calculated at baseline/initial diagnosis (baseline was the first day of admission for patients who met HBV-ACLF criteria at admission; for those who didn't, baseline was the day of diagnosis) and on the final day (defined as the last day of the 28-day follow-up period or the last examination before death or discharge from the hospital). Prognostic scores, including model for end-stage liver disease (MELD) score (14), the chronic liver failure-sequential organ failure assessment (CLIF-SOFA) (1), CLIF Consortium Organ Failure score (CLIF-C OFs), CLIF Consortium ACLF score (CLIF-C ACLFs) (7), COSSH-ACLFs (2), and COSSH-ACLF IIs were calculated at diagnosis. Survival information at days 28 and 90 was collected via medical records, telephone interviews, or outpatient visits after discharge.

### Definitions

The APASL criteria for ACLF are an acute hepatic insult manifesting as jaundice (serum bilirubin ≥ 5 mg/dL) and coagulopathy (INR ≥ 1.5 or prothrombin activity < 40%) complicated with clinical ascites and/or hepatic encephalopathy (HE) within 4 weeks in a patient with previously diagnosed or undiagnosed chronic liver disease/cirrhosis (8). According to the updated proposals (15, 16), when the above criteria were met, patients with a history of decompensated cirrhosis were also included.

The definitions of DIC score, organ failure, infection, ascites grade, cirrhosis, COSSH-ACLF grades, EASL-ACLF grades, clinical course, chronic hepatitis B, HBV reactivation, as well as MELD, CLIF-C OFs, CLIF-C ACLFs, COSSH-ACLFs, and CLIF-SOFA score are described in Supplementary Method 2.

All patients received standard medical treatment (detailed information is given in Supplementary Method 3).

### Statistical Analyses

All statistical analyses were performed using Graphpad 8.0 (Graphpad Software, San Diego, CA) and SPSS version 23 (SPSS, Chicago, IL). Categorical variables were expressed as percentages (frequencies), and continuous variables were expressed as medians (interquartile ranges). Categorical variables were compared using the Chi-squared test or Fisher's test. Continuous variables were analyzed using the Mann-Whitney *U*-test, the Kruskal-Wallis test, or the Wilcoxon matched-pairs signed rank test, as appropriate. The association between DIC score system and MELD score was assessed using Pearson correlation coefficient. Survival probabilities based on DIC scores and CLIF-C OF-DIC scores at diagnosis were estimated using Kaplan-Meier survival curves and compared using the log-rank test. The auROC and Z-test (Delong's method) were used to compare the predictive value of different prognostic scoring models. A multivariate Cox regression analysis was performed to identify independent prognostic factors for HBV-ACLF according to the enter method. Two-tailed *P*-values were calculated, and the significance level was set at *P* < 0.05.

## Results

### Patient Characteristics

This study included 163 patients diagnosed with HBV-ACLF according to the APASL criteria, of which 73 died within 90 days (Figure 1). The clinical characteristics of all enrolled patients according to their 90-day survival states are summarized in Table 1. The most common precipitating event was hepatitis B relapse (*n* = 78, 47.9%), followed by bacterial infection (*n* = 9, 5.5%). Moreover, liver failure was the most frequent type of organ failure (*n* = 136, 83.4%), followed by coagulation failure (*n* = 61, 37.4%). Compared to survivors, patients who died within 90 days had more complications, of which 61 (83.6%) presented with bacterial infection, 61 (83.6%) suffered from ascites, and 11 (15.1%) had complications with gastrointestinal (GI) hemorrhage. Survivors had higher alanine aminotransferase, sodium, platelet, and hemoglobin levels but had significantly lower DIC scores, INR, and occurrence of organ failure (except liver failure) and were younger.

**Table 1:** Clinical characteristics of patients with HBV-ACLF.

	**Total cohort (*n* = 163)**	**Survivors (*n* = 90)**	**No-survivors (*n* = 73)**	***P*-value**
**Clinical data**				
Age (yr)	46 (37–56)	43 (33–53)	51 (42–62)	0.000
Male sex, % (no.)	91.4 (149)	92.2 (83)	90.4 (66)	0.682
Underlying liver disease, % (no.)				0.101
Chronic hepatitis B	49.1 (80)	55.6 (50)	41.1 (30)	
Compensated cirrhosis	31.3 (51)	30.0 (27)	32.9 (24)	
Decompensated cirrhosis	19.6 (32)	14.4 (13)	26.0 (19)	
Precipitating events				0.135
HBV reactivation, % (no.)	47.9 (78)	56.6 (51)	37.0 (27)	
Bacterial infection, % (no.)	5.5 (9)	3.33 (3)	8.2 (6)	
Superimposed HAV or HEV infection, % (no.)	3.7 (6)	4.4 (4)	2.7(2)	
Hepatotoxic drugs, % (No.)	4.9 (8)	4.4 (4)	5.5 (4)	
Active drinking, % (No.)	4.3 (7)	4.4 (4)	4.1 (3)	
Unknown, % (No.)	33.7 (55)	26.6 (24)	42.5 (31)	
**Complications, % (no.)**				
Ascites	69.3 (113)	57.8 (52)	83.6 (61)	0.000
GI hemorrhage	7.4 (12)	1.1 (1)	15.1 (11)	0.001
Bacterial infection	62.0 (101)	44.4 (40)	83.6 (61)	0.000
Artificial liver, % (no.)	28.2 (46)	20.0 (18)	38.4 (28)	0.010
**Laboratory data**				
Alanine aminotransferase (U/L)	157 (72–431)	184 (92–467)	150 (57–341)	0.059
Albumin (g/L)	32 (29–36)	32 (29–36)	32 (29–37)	0.507
Total bilirubin (μmol/L)	307.1 (72.0–431.0)	284.5 (220.5–358.0)	354.0 (257.1–503.8)	0.000
Creatinine (μmol/L)	69 (58–86)	68 (57–75)	75 (59–109)	0.006
Sodium (mmol/L)	136 (132–139)	137 (134–140)	135 (130–138)	0.004
White blood cell count (10^9^/L)	6.76 (5.04–10.36)	6.24 (4.51–8.37)	8.29 (5.79–10.74)	0.005
Hemoglobin (g/L)	120 (104–136)	123 (109–139)	117.5 (94–130.5)	0.024
Platelet count (10^9^/L)	90 (62–121)	103 (79–133)	76 (47–101)	0.000
INR	2.11 (1.80–2.64)	1.85 (1.71–2.11)	2.65 (2.25–3.19)	0.000
DIC score	5 (4–6)	4 (4–5)	6 (5–7)	0.000
Fibrinogen (g/L)	1.17 (0.8–1.5)	1.3 (1–1.7)	0.91 (0.62–1.30)	0.000
D-dimer (mg/L FEU)	3.05 (1.42–4.92)	1.73 (0.95–3.66)	4.25 (2.91–9.54)	0.000
FDPs (mg/L)	7.5 (3.6–14.6)	4.1 (2.6–9.6)	12.9 (7.4–32.6)	0.000
**Organ failure, % (no.)**				
Liver	83.4 (136)	78.9 (71)	89.0 (65)	0.083
Coagulation	37.4 (61)	8.9 (8)	72.6 (53)	0.000
Kidney	14.1 (23)	4.4 (4)	26.0 (19)	0.000
Cerebral	19.6 (32)	0 (0)	43.8 (32)	0.000
Lung	8.6 (14)	0 (0)	19.2 (14)	0.000
Circulation	14.61(23)	0 (0)	31.5 (23)	0.000

*The information about ascites, laboratory data was acquired at baseline while GI hemorrhage, bacterial infection, artificial liver treatment, organ failure assessment was gathered throughout the whole course within 90 days*.

*ACLF, acute-on-chronic liver failure; HBV, hepatitis B virus; INR, international normalized ratio; COSSH-ACLF, Chinese Group on the Study of Severe Hepatitis B-ACLF; GI, gastrointestinal; DIC, disseminated intravascular coagulation; FDPs, fibrinogen degradation products; HAV, hepatitis A virus; HEV, hepatitis E virus*.

*Data are expressed as the median (interquartile range) or percent (number)*.

We graded patients according to EASL-ACLF criteria and COSSH-ACLF criteria. According to EASL-ACLF criteria, 103 patients had ACLF grade 0 (63.19%), 3 patients ACLF grade 1 (1.84%), 47 patients ACLF grade 2 (28.83%), and 10 patients ACLF grade 3 (6.13%) at baseline. In Europe, the first two causes of ACLF were alcohol and hepatitis C; alternately, in the Asian-Pacific and African regions, HBV infection was the main cause of ACLF. Patients with different etiologies vary in clinical features. COSSH-ACLF criteria were proposed based on a prospective multicenter cohort of HBV-ACLF patients (2). According to COSSH-ACLF criteria, 21 patients was ACLF grade 0 (12.88%), 85 patients ACLF grade 1 (52.15%), 47 patients ACLF grade 2 (28.83%), and 10 patients ACLF grade 3 (6.13%) (Supplementary Figure 1).

### All Indicators in the DIC Score System Were Correlated With Poor Prognosis

**Figure 2:**
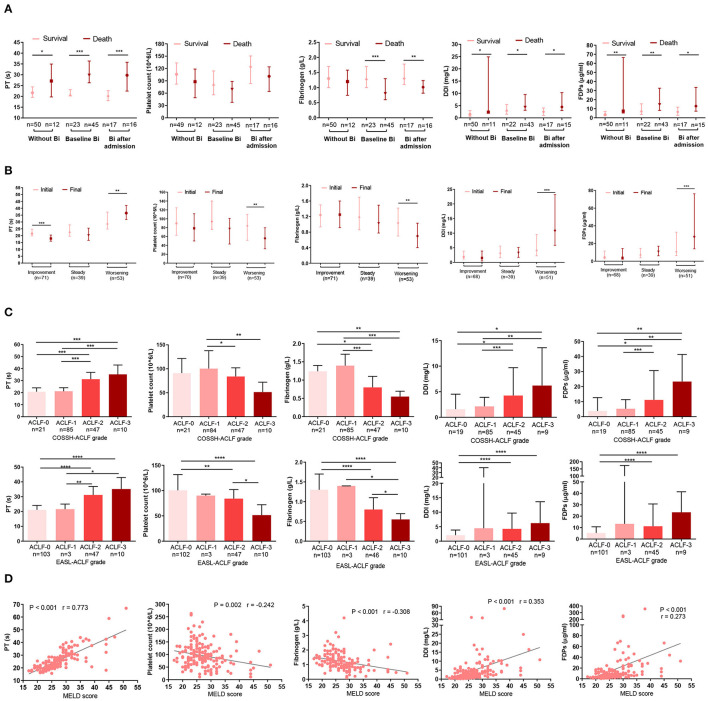
Association between indicators in the DIC score system and prognosis. **(A)** The bar indicates the mode of DIC score indicators (platelet count, PT, DDI, FDPs, and fibrinogen) in infected or non-infected HBV-ACLF patients who survived or died. Without Bi: patients who never had infection during the whole course within 90 days; baseline Bi: patients who had infection at baseline; Bi after admission: patients without infection at baseline and developed bacterial infection within 90 days. **(B)** Changes in DIC score indicators (platelet count, PT, DDI, FDPs, and fibrinogen) according to the clinical course. Initial: the parameters were detected at baseline; final: the parameters were detected at last within 28 days of diagnosis or before death or discharge from the hospital. **(C)** Association between DIC score indicators (platelet count, PT, DDI, FDPs, and fibrinogen) and COSSH-ACLF/EASL-ACLF grade. **(D)** Correlations between DIC score indicators (platelet count, PT, DDI, FDPs and fibrinogen) and MELD score. Platelet count was missing in one patient, and five patients lacked D-dimer and FDPs information. Some patients had been treated in other hospitals before being admitted to Huashan Hospital, so some had complications with Bi upon admission. DIC, disseminated intravascular coagulation; PT, prothrombin time; DDI, D-dimer; FDPs: fibrinogen degradation products; HBV-ACLF, hepatitis B virus-related acute-on-chronic liver failure; Bi, bacterial infection; COSSH-ACLF, Chinese Group on the Study of Severe Hepatitis B-acute-on-chronic liver failure; EASL-ACLF, European Association for the Study of the Liver-ACLF; MELD, Model for end-stage liver disease. ^*^P < 0.05, ^**^P < 0.01, and ^***^P < 0.001.

The DIC scoring system includes platelet count, a fibrin-related marker, fibrinogen, and PT. We assessed these parameters at baseline and on the final day. Patients with infection at baseline had worse DIC score system than those without infection or those that developed infection after admission (Supplementary Figure 2). More importantly, however, was that in both bacteria-infected and uninfected patients, PT, D-dimer, and fibrinogen degradation products (FDPs) levels were significantly higher in non-survivors than survivors. Conversely, fibrinogen and platelet count were higher in survivors (Figure 2A).

The dynamic changes in DIC indicators were consistent with the clinical course. Among the HBV-ACLF patients, the clinical course improved in 71, worsened in 53, and was steady in 39. Compared with the initial day, patients with a worsening clinical course on the final day had longer PTs (*P* = 0.004) and higher D-dimer (*P* < 0.001) and FDPs (*P* < 0.001) levels. However, their platelet counts (*P* = 0.005) and fibrinogen (*P* = 0.004) levels were lower (Figure 2B). Similarly, ACLF-3/-2 patients had prolonged PT and increased D-dimer and FDPs levels, as well as reduced fibrinogen and platelet counts than ACLF-1/-0 patients (Figure 2C). Finally, we analyzed the relationship between index in the DIC scoring system and MELD score. MELD scores were positively associated with D-dimers (*r* = 0.353, *P* < 0.001), FDPs (*r* = 0.273, *P* < 0.001), and PT (*r* = 0.773, *P* < 0.001) but were inversely correlated with fibrinogen (*r* = −0.308, *P* < 0.001) and platelet counts (*r* = −0.242, *P* = 0.002) (Figure 2D). Note that platelet count was missing in one patient, and five patients lacked information on D-dimer and FDPs.

### DIC Score Were Correlated With Poor Prognosis

Considering that all DIC indicators were significantly correlated with survival, clinical course, and disease severity, we further evaluated the prognostic value of the DIC score in HBV-ACLF.

**Figure 3:**
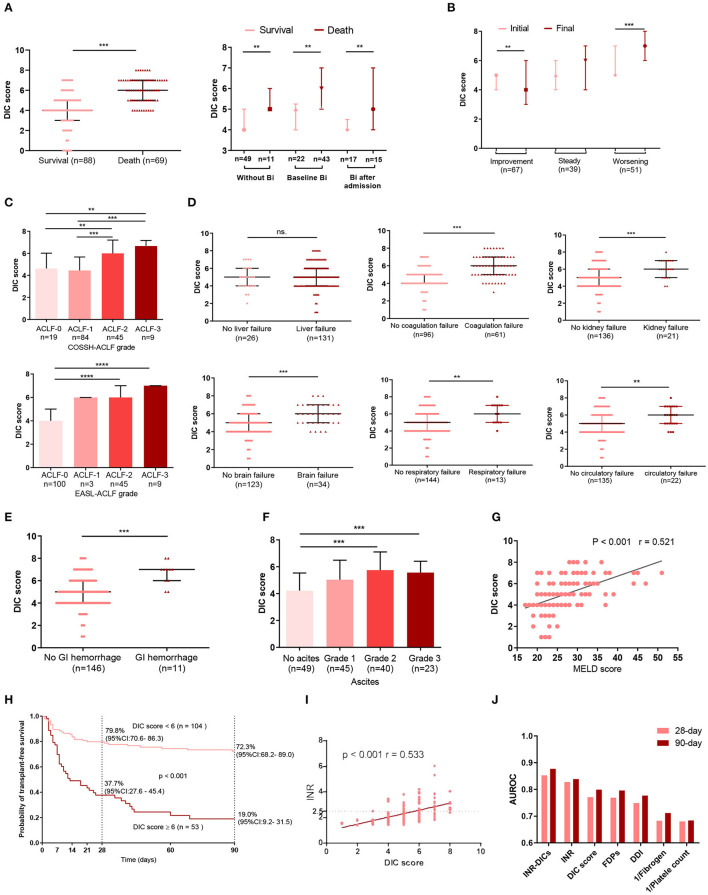
The relationship between complications, organ failure, prognosis, INR and DIC score. **(A)** The DIC scores of ACLF patients with or without bacterial infection were sorted by survival states. Without Bi: patients who never had infection during the whole course within 90 days; Baseline Bi: patients who had infection at baseline; Bi after admission: patients without infection at baseline and developed bacterial infection within 90 days. **(B)** Dynamic changes in DIC score from initial to final assessment according to clinical course. Initial: the DIC score was assessed at baseline; Final: the last DIC score assessed within 28 days of diagnosis or before death or discharge from the hospital. **(C)** Comparisons of DIC scores among subgroups of ACLF patients according to COSSH-ACLF, and EASL-ACLF grade. The association between DIC scores and single organ failure is illustrated in **(D)**. **(E)** Comparison of DIC scores between patients with or without GI. **(F)** The relationship between ascites and DIC scores. **(G)** Correlations between DIC score and MELD score. **(H)** 28- and 90-day transplant-free survival rates of HBV-ACLF patients based on the cutoff DIC score at diagnosis. **(I)** The correlation between DIC score and INR. **(J)** The area under the receiver operating curve of the DIC score system at diagnosis for predicting the 28-day and 90-day mortality of HBV-ACLF patients. For 28-day mortality: INR-DICs, 0.853; INR, 0.828; DIC score, 0.771; FDPs, 0.769; DDI, 0.749; Fibrogen, 0.683; platelet, 0.680. For 90-day mortality: INR-DICs, 0.877; INR, 0.839; DIC score, 0.799; FDPs, 0.796; DDI, 0.777; 1/Fibrogen, 0.712; 1/platelet, 0.684. DIC, disseminated intravascular coagulation; ACLF, acute-on-chronic liver failure; BI, bacterial infection; COSSH-ACLF, Chinese Group on the Study of Severe Hepatitis B-acute-on-chronic liver failure score; GI, gastrointestinal hemorrhage; MELD, Model for End-stage Liver Disease; INR, the international normalized ratio; PT, prothrombin time; DDI, D-dimer; FDPs, fibrinogen degradation products; INR-DICs, INR combined with DIC score; HBV-ACLF, hepatitis B virus-related acute-on-chronic liver failure; EASL-ACLF, European Association for the Study of the Liver-ACLF. ^*^P < 0.05, ^**^P < 0.01, and ^***^P < 0.001.

In both bacteria-infected and uninfected groups, DIC score was significantly higher in non-survivors than survivors (Figure 3A), and the DIC scores of patients with infection were higher than those without (Supplementary Figure 2). Also, patients with a worsening clinical course on the final day had higher DIC scores (*P* < 0.001) than on the initial day, and patients with an improving clinical course presented with lower DIC scores on the final day (*P* = 0.008; Figure 3B). Patients with more organ failures (ACLF-3/-2) had higher DIC scores (Figure 3C), as did those with complications such as organ failure (coagulation, kidney, cerebral, lungs, circulatotion) (Figure 3D), or GI hemorrhage (Figure 3E). Previous studies have reported that ascites contains large amounts of fibrinolytic products (17); accordingly, we found that patients with grade 2 or 3 ascites had larger DIC scores (Figure 3F). MELD scores were also positively associated with DIC scores (*r* = 0.521, *P* < 0.001; Figure 3G).

The auROC of the DIC scores for 90-day mortality was 0.799 with a sensitivity of 0.594 and specificity of 0.864 at a cut-off value of 6. Patients with DIC score< 6 at diagnosis had significantly improved short- and mid-term survival than those with DIC scores ≥ 6 (28 day: 79.8 vs. 37.7%, 90-day: 72.3 vs. 19.0%, *P* < 0.001) (Figure 3H).

Although DIC score performs well in predicting prognosis in HBV-ACLF, the majority of ACLF patients (*n* = 142, 87.1%) had PT extensions of more than 6 sec (PT > 6 sec are scored two points in the DIC score), so their DIC scores were mainly affected by the other three indicators (platelet counts, D-dimer and fibrinogen levels). INR, as the most widely used indicator of ACLF prognosis, is mathematically standardized PT. Thus, combining INR with DIC score may be more accurate in reflecting the status of the coagulation system in HBV-ACLF patients. Besides, *r* value of Pearson correlation between the INR and DIC scores was 0.533 (Figure 3I). To assess the prognosis predictive value of the combination of INR and DIC scores, we put DIC score and INR into a multivariate Cox regression (Supplementary Table 1), and obtained a new coagulation score for HBV-ACLF patients (INR-DIC score) as calculated by the following formula: INR-DICs = 0.404 × DIC score + 0.916 × INR. Then we calulated the auROC of INR-DIC and individual makers of DIC score, and found that combined INR with DIC score had the largest auROC (28-day prognosis: 0.853; 90-day prognosis: 0.877) (Figure 3J).

### Development of a Prognostic Score for HBV-ACLF

We performed a multivariate Cox regression to identify the most significant factors related to survival (Supplementary Table 2). We found age (hazard ratio [HR]: 1.031, 95% confidence interval [CI]: 1.005–1.056, *P* = 0.017), INR (HR: 2.524, 95% CI: 1.628–3.914, *P* = 0.000), HE grade (HR: 1.494, 95% CI: 1.117–1.999, *P* = 0.007), total bilirubin (HR: 1.001, 95% CI: 1.001–1.002, *P* = 0.000), and DIC scores (HR: 1.397, 95% CI: 1.040–1.875, *P* = 0.026) to be independent risk factors.

Among the identified independent predictors of death, INR, total bilirubin, and hepatic encephalopathy are components of CLIF-C OFs that are used to assess organ failure. CLIF-C OFs is a classic prognosis model widely used in ACLF. Therefore, to improve the prognostic value of CLIF-C OFs, a multivariate Cox regression including CLIF-C OFs, DIC scores and age was analyzed again (Table 2). We obtained a new prognostic score for HBV-ACLF patients (CLIF-C OF-DIC score) as calculated by the following formula: CLIF-C OF-DICs = 0.679 × CLIF-C OFs + 0.344 × DIC score + 0.039 × Age. The auROC of the CLIF-C OF-DICs for 90-day mortality was 0.936 with a sensitivity of 84.06% and specificity of 88.64% at a cut-off value of 10.03.

**Table 2:** Risk factors associated with transplant-free 90-day mortality in patients with HBV-ACLF according to a multivariate Cox PH model.

	**Regression coefficient**	**HR**	**95%CI**	***P*-value**
Age (yr)	0.039	1.039	1.018–1.061	0.000
DIC score	0.344	1.410	1.148–1.733	0.000
CLIF-C OFs	0.679	1.971	1.680–2.312	0.000

*ACLF, acute-on-chronic liver failure; HBV, hepatitis B virus; CLIF-C OFs, Chronic liver failure-consortium organ function score; DIC, disseminated intravascular coagulation*.

**Figure 4:**
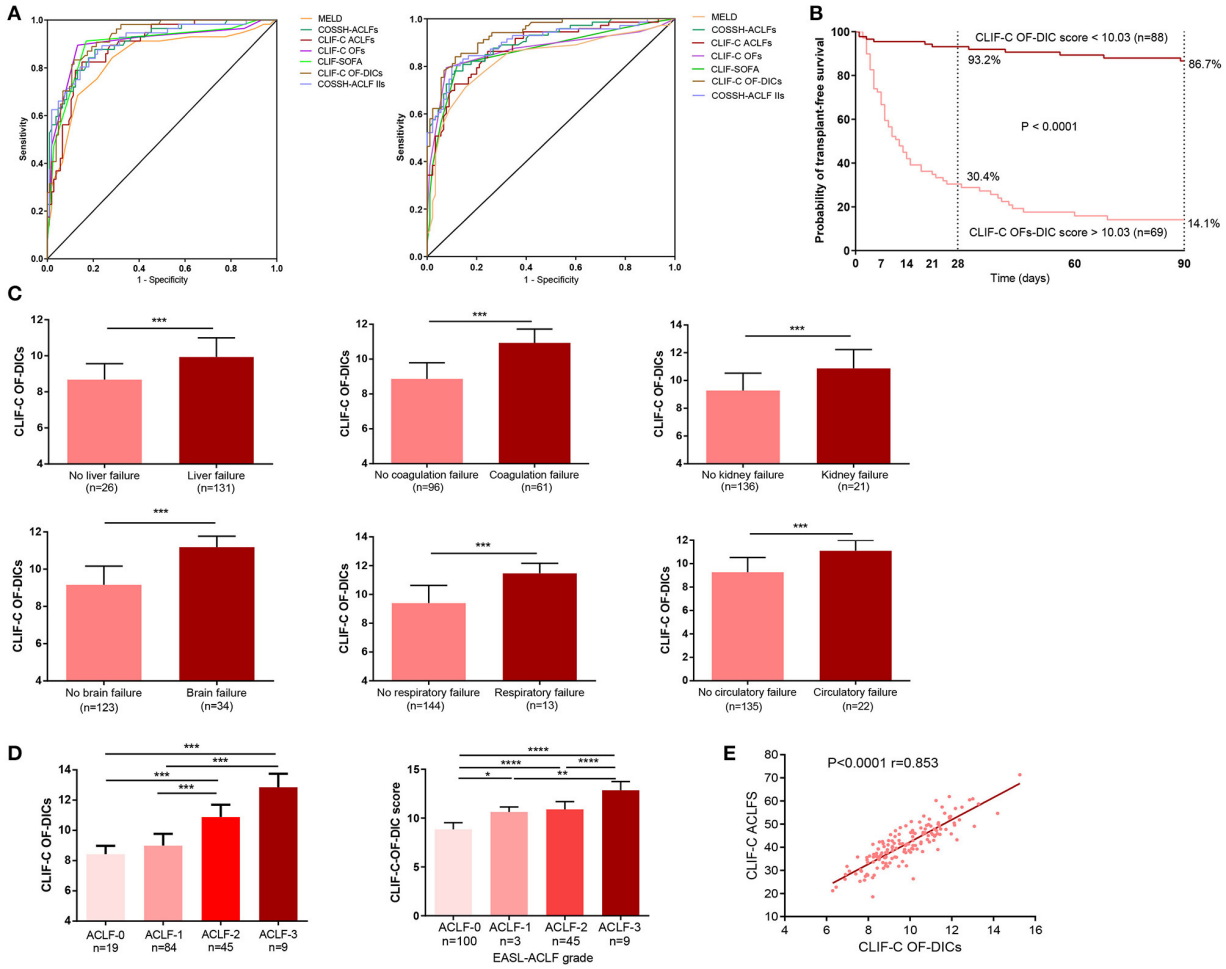
Seven prognostic models used to predict the 28- and 90-day mortality of patients and the correlation between CLIF-C OF DICs and disease severity. **(A)** Accuracy of the CLIF-OF C-DICs as compared to MELDs, COSSH-ACLFs, CLIF-C ACLFs, CLIF-C OFs, CLIF-SOFA, and COSSH-ACLF-IIs in predicting 28-day (left) and 90-day (right) mortality of ACLF patients. The areas under the receiver operating curve were as follows. For 28-day mortality: CLI-C OF-DICs, 0.924; CLIF C-OFs, 0.900; CLIF-C ACLFs, 0.888; CLIF-SOFA, 0.895; MELD, 0.836; COSSH-ACLFs, 0.904; COSSH-ACLF-IIs, 0.905. For 90-day mortality: CLI-C OF-DICs: 0.936; CLIF C-OFs, 0.870; CLIF-C ACLFs, 0.879; CLIF-SOFA, 0.863; MELD, 0.830; COSSH-ACLFs, 0.903; COSSH-ACLF-IIs, 0.901. **(B)** Probability of 28- and 90-day transplant-free survival in ACLF patients based on the CLIF-C OF-DICs cutoff value (10.03). Kaplan-Meier curves were compared using the log-rank test. **(C)** The CLIF-C OF-DICs between patients with organ failure or not. **(D)** The CLIF-C OF-DICs in patients with different ACLF grades. **(E)** The correlation between CLIF-C OF-DICs and CLIF-C ACLFs. ACLF, acute-on-chronic liver failure; DIC, disseminated intravascular coagulation; CLIF, Chronic Liver Failure; COSSH-ACLF, Chinese Group on the Study of Severe Hepatitis B-ACLF; EASL-ACLF, European Association for the Study of the Liver-ACLF; CLIF-SOFA score, CLIF-sequential organ failure assessment score; CLIF-C OF-DICs, a novel prognostic score based on age, DIC score, and CLIF-C OFs; MELD, Model for end-stage liver disease; COSSH-ACLFs, Chinese Group on the Study of Severe Hepatitis B-ACLF score; CLIF-C OFs, CLIF-Consortium Organ Failure score; CLIF-C ACLFs, CLIF-Consortium ACLF score; COSSH-ACLF IIs, Chinese Group on the Study of Severe Hepatitis B-ACLF II score. ^*^P < 0.05, ^**^P < 0.01, and ^***^P < 0.001.

We compared the prognostic value of CLIF-C OF-DICs for 28- and 90-day mortality with MELD, CLIF-SOFA, CLIF-C ACLFs, CLIF-C OFs, COSSH-ACLFs, and COSSH-ACLF-IIs and found the CLIF-C OF-DICs had the highest auROC (28-day: 0.924, 90-day: 0.936; Figure 4A and Supplementary Table 3). Using the cut-off value, patients in the derivation cohort were categorized into the high CLIF-C OF-DICs (CLIF-C OF-DICs > 10.03) and low CLIF-C OF-DICs (< 10.03) groups. The cumulative 28- and 90-day transplant-free survival rates of the low CLIF-C OF-DICs group were significantly higher than the high CLIF-C OF-DICs group (28-day: 93.2 vs. 30.4%, 90-day: 86.7 vs. 14.1%; *P* < 0.0001; Figure 4B). Besides, patients with organ failures (liver, coagulation, kidney, cerebral, lungs, circulation) had higher CLIF-C OF-DICs (Figure 4C), as did those with higher ACLF grade (Figure 4D). CLIF-C ACLFs scores were also positively associated with CLIF-C OF-DICs (*r* = 0.853, *P* < 0.001; Figure 4E).

### External Validation of DIC Score Performance

We recruited an external cohort to validate the prognostic value of DIC score and CLIF-C OF-DICs. The clinical and laboratory characteristics of the derivation and validation cohorts are listed in Supplementary Table 4. The two cohorts differed on 28-day mortality, their distributions of underlying liver diseases, and baseline laboratory data, which included total bilirubin, creatinine, fibrinogen, and D-dimer levels. DIC score demonstrated similar prognostic value in an external validation group of 82 HBV-ACLF patients.

**Figure 5:**
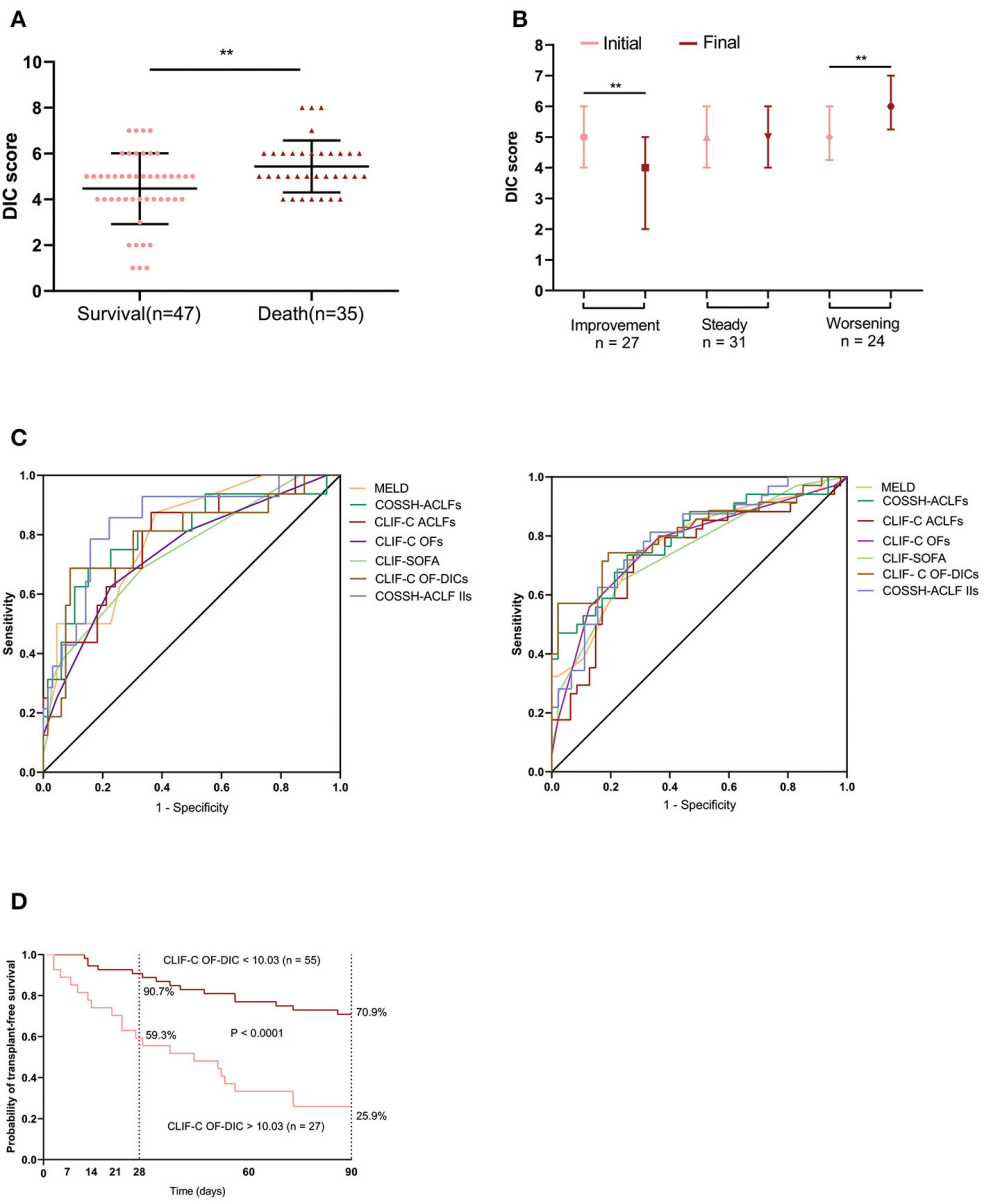
DIC scores in external HBV-ACLF validation cohort. **(A)** The bars indicate DIC scores between patients who survived or died within 90 days. **(B)** Changes in DIC scores from initial to final assessments according to clinical course. Initial: the DIC score was assessed at baseline; final: the last DIC score assessed within 28 days of diagnosis, or before death or discharge from the hospital. **(C)** Comparison of CLIF-C OF-DICs with MELD, COSSH-ACLFs, CLIF-C ACLFs, CLIF-C OFs, CLIF-SOFA and COSSH-ACLF-IIs in predicting 28-day and 90-day mortality of ACLF patients. The areas under the receiver operating curve were as follows. For 28-day mortality: CLI-C OF-DICs, 0.791; CLIF C-OFs, 0.744; CLIF-C ACLFs, 0.784; CLIF-SOFA, 0.744; MELD, 0.804; COSSH-ACLFs, 0.804; COSSH-ACLF-IIs, 0.845. For 90-day mortality: CLI-C OF-DICs: 0.812; CLIF C-OFs, 0.767; CLIF-C ACLFs, 0.737; CLIF-SOFA, 0.741; MELD, 0.774; COSSH-ACLFs, 0.797; COSSH-ACLF-IIs, 0.790. **(D)** Probability of 28- and 90-day transplant-free survival in ACLF patients based on the CLIF-C OF-DICs cutoff value (10.03) acquired in the derivation cohort. DIC, disseminated intravascular coagulation; ACLF, acute-on-chronic liver failure; HBV-ACLF, hepatitis B virus-related ACLF; CLIF, Chronic Liver Failure; CLIF-SOFA, CLIF-sequential organ failure assessment score; CLIF-C OF-DICs, a novel prognostic score based on age, DIC score, and CLIF-C OFs; MELD, Model for end-stage liver disease; CLIF-C ACLFs, CLIF-Consortium ACLF score; CLIF-C OFs, CLIF-Consortium Organ Failure score; COSSH-ACLFs, Chinese Group on the Study of Severe Hepatitis B-ACLF score; COSSH-ACLF IIs, Chinese Group on the Study of Severe Hepatitis B-acute-on-chronic liver failure II score. ^*^P < 0.05, ^**^P < 0.01, and ^***^P < 0.001.

Compared with survivors, non-survivors had significantly higher DIC scores (*P* = 0.006, Figure 5A). The association between DIC scores and clinical course is illustrated in Figure 5B. DIC scores declined in HBV-ACLF patients with an improving clinical course (*P* = 0.002), remained nearly constant in patients with a steady clinical course (*P* = 0.364), and significantly increased in patients with a worsening clinical course (*P* = 0.001).

The prognostic value of CLIF-C OF-DICs in predicting 28-day mortality was comparable to six other generic prognostic score models (auROC: 0.791) and was superior in predicting 90-day mortality (auROC: 0.812) in HBV-ACLF patients, although this increase was not statistically significant (Figure 5C). The auROC of all scores are listed in Supplementary Table 5. The cumulative survival rates of the low CLIF-C OF-DICs group (< 10.03) were significantly higher than the high CLIF-C OF-DICs group (> 10.03) with 28-day survival rates of 90.7% and 59.3% and 90-day survival rates of 70.9% and 25.9%, respectively (*P* < 0.0001, 90-day mortality: HR: 0.255, 28-day mortality: HR: 0.184; Figure 5D).

## Discussion

This retrospective study evaluated the DIC score system in patients with HBV-ACLF and demonstrated that these subjects frequently displayed an abnormal coagulation state similar to DIC. They had prolonged PT, reduced fibrinogen, decreased platelet counts, elevated D-dimers and FDPs, and increased overall DIC scores. DIC scores system deteriorates as ACLF grade increases, and the results were consistent under different diagnostic criteria (EASL or COSSH ACLF criteria). A prolonged PT, a result of impaired liver synthesis function, has been widely recognized in ACLF patients (2). Thrombocytopenia is common in patients with advanced cirrhosis, and it is related to portal hypertension and hepatic decompensation (18). Fibrinogen, a key component of blood clots and a modest acute-phase reactant, is primarily synthesized in hepatocytes and has a shorter half-life in cirrhosis (3, 19). Fisher et al. discovered that compared to healthy controls, patients with acute decompensated cirrhosis and ACLF have lower fibrinogen levels, which in stable cirrhosis patients are a bit higher (5). The abnormally elevated D-dimer and FDPs levels in ACLF patients reflect activated coagulation and fibrinolysis, which is consistent with other studies in patients with cirrhosis and ACLF (6, 20–22). The origin of the elevated D-dimer and FDP levels is not clear. Ascites may be a source of fibrinolytic products (17), which was common in our cohort accounting for 69.3% of patients and was associated with DIC score. However, at present, no evidence of pathological microthrombosis in ACLF has been reported.

The widely activated coagulation system in HBV-ACLF patients may result from infection or a high-inflammatory state (23). Infection and sepsis are prevalent in ACLF patients and are related to their prognosis (24, 25). Up to 62.0% of patients in our cohort had bacterial infections. Hemostatic changes in ACLF and sepsis greatly overlap: they are both associated with elevated levels of VWF, D-dimer, factor VIII, thrombin-antithrombin and decreased levels of the VWF-regulating protease ADAMTS13 and coagulation factors (6). Hypercoagulable profiles and hypofibrinolysis states result in microvascular thrombosis or even DIC; they play essential roles in the pathological process of multiple organ failure in sepsis (26). Therefore, changes in the hemostasis system of ACLF patients result from multiple complex factors, which include the impairment of liver synthesis function, the consumption of coagulation factors in intravascular coagulation, and the accumulation of secondary fibrinolytic products. Furthermore, infection and systemic inflammatory response syndrome may aggravate coagulation disorders (23, 27). These results emphasize the importance of coagulation and fibrinolysis in the progression of ACLF and provide ideas for follow-up studies in their mechanisms.

The DIC score was proposed by the ISTH in 2001 to standardize DIC criteria (10). It has been applied in predicting the prognosis of sepsis, post-trauma multiple organ dysfunction syndrome, and thrombosis in acute myeloid leukemia (12, 28, 29). Here, we demonstrated that the DIC score, as an independent predictor of 90-day mortality, forms part of a novel prognostic tool in patients with HBV-ACLF. The DIC score is more comprehensive in the evaluation of coagulation, but in the DIC score, the upper limit for evaluating the PT is extended by 6 s, and most ACLF patients have a PT extension of more than 6 s. INR is the mathematical standardization of PT. Therefore, DIC score combined with INR is more effective in assessing coagulation dysfunction and prognosis in hepatitis B virus acute-on-chronic liver failure patients.

Because HBV-ACLF can deteriorate rapidly and lead to death, an accurate prognostic score can help liver transplantation decision making. CLIF-C OFs is a summation of organ failure severity that is used for ACLF diagnosis and prognosis prediction (1). To improve CLIF-C OFs, we combined DIC score with age and CLIF-C OFs to generate a new prognostic model, CLIF-C OF-DICs, and we confirmed its predictive power in derivation and validation cohorts. These two cohorts differed in clinical characteristic and auROC, which may be related to the different patient sources and treatment strategies between the two hospitals, as well as the patients' economic situations.

This study had several limitations. The DIC score analysis and the CLIF-C OF-DICs prognostic model were based on a retrospective analysis, and the sample size was relatively low. Moreover, the patients in the current cohort were HBV-related, and the prognosis value of DIC scores in ACLF patients associated with other etiologies need to be determined. In the future, additional prospective studies with larger patient populations should be conducted to clarify the DIC score system in ACLF patients and to explore the mechanisms underlying coagulation and fibrinolysis disorders.

In conclusion, this study demonstrated that DIC scores were correlated with short-term prognosis in HBV-ACLF. Patients with elevated DIC scores (≥6) at admission had increased risks of 28- and 90-day mortality. Monitoring the DIC score will help in predicting short-term prognosis in HBV-ACLF patients.

## Data Availability Statement

The original contributions presented in the study are included in the article/Supplementary Material, further inquiries can be directed to the corresponding author/s.

## Ethics Statement

The studies involving human participants were reviewed and approved by the Ethical Committee of Huashan Hospital of Fudan University and the Ethical Committee of the Shanghai Public Health Clinical Center. The patients/participants provided their written informed consent to participate in this study.

## Author Contributions

YH and XZ made the study concept and design, statistical analysis and drafting of manuscript was done by JH and XZ. The collection of data and statistics for patient recruitment in the verification cohort was completed by XQ. The data collection of the deriving cohort by YZ and XL. Enrolling participants for deriving cohort was done by YY and PZ. Critical revision of manuscript done for important intellectual content was done by JZ and WZ. All authors contributed to the article and approved the submitted version.

## Funding

This work was supported by National Natural Science Foundation of China (Nos. 81670560, 81871640, and 82172255), National Science and Technology Major Project (2017ZX09304005 and 2017ZX10202202), Shanghai Shen Kang Hospital Development Center (Nos. SHDC12021114 and SHDC12019116), and Shanghai Key Clinical Specialty Construction Program (ZK2019B24).

## Conflict of Interest

The authors declare that the research was conducted in the absence of any commercial or financial relationships that could be construed as a potential conflict of interest.

## Publisher's Note

All claims expressed in this article are solely those of the authors and do not necessarily represent those of their affiliated organizations, or those of the publisher, the editors and the reviewers. Any product that may be evaluated in this article, or claim that may be made by its manufacturer, is not guaranteed or endorsed by the publisher.
