# Supplementary material for: DIC Score Combined With CLIF-C OF Score Is More Effective in Predicting Prognosis in Patients With Hepatitis B Virus Acute-on-Chronic Liver Failure
Source: Front Med (Lausanne). 2022 Feb 11;9:815580. doi: 10.3389/fmed.2022.815580 (PMC8878906; doi:10.3389/fmed.2022.815580)
Supplement: Supplementary file 1 [file Data_Sheet_1.docx]

**Supplementary materials:**

**Contents of Supplementary data**

| **contents** | **number** |
| --- | --- |
| **Supplementary methods** | **3** |
| **Supplementary Tables** | **5** |
| **Supplementary Figure** | **2** |

**Methods S1:**

**Inclusion criteria (only patients who meet the following 3 items can be admitted):**

1. Hospitalized patient ( > 1 day，including patients hospitalized at ER for observation).
2. APASL criteria for ACLF: Patients with an acute hepatic insult manifesting as jaundice (serum bilirubin ≥ 5 mg/dL (85 micro mol/L) and coagulopathy (INR ≥ 1.5 or prothrombin activity < 40%) complicated within 4 weeks by clinical ascites and/or encephalopathy in a patient with previously existed diagnosed or undiagnosed chronic liver disease. Patients with known previous decompensation cirrhosis was included as well. (1)
3. The underlying liver disease was chronic hepatitis B (including chronic hepatitis B without cirrhosis, chronic hepatitis B-related compensated cirrhosis and chronic hepatitis B-related decompensated cirrhosis).

**Exclusion criteria: (patients who meet any one of the following 8 items should be excluded):** (2)

1. Patients aged <18 or >80 years
2. Patients who have had hepatocellular carcinoma or other liver malignancies
3. Patients who have had any other tumors
4. Patients who have had severe extrahepatic disease: (a) COPD combined with respiratory failure; (b) Coronary heart disease with 3-level cardiac function; (c) Myocardial infarction in the 3 months before hospitalization; (d) failure of renal function due to a chronic renal disease. (e) serious complications of diabetes (causing damage to heart, brain, kidney, feet, etc.)
5. Patients receiving immunosuppressive drugs for reasons other than chronic liver diseases (with a year): (a) Chemotherapy against tumor; (b) Therapy against connective disease in the active phase; (c) Nephrotic syndrome.
6. Patients who complicated with chronic liver disease of other etiology: Alcoholic hepatitis; chronic hepatitis C; non-alcoholic fatty liver disease; autoimmune hepatitis; schistosomiasis; Wilson’s; drug induced liver disease.
7. Patients who are pregnant.
8. Patients taking anticoagulants or platelet aggregation inhibitors.
9. Patients received liver transplatation within 28 days;
10. Patients lost to follow-up within 28 days.

**Methods S2:**

**Definition for DIC score, organ failure, infection, cirrhosis, ACLF grade, clinical course, chronic hepatitis B, HBV reactivation, various prognosis models:**

**DIC score:** The DIC score is a diagnostic scoring system for DIC proposed by the International Society for Thrombosis and Haemostasis (ISTH). It includes platelet count (> 100 × 109/L = 0; < 100 × 109/L = 1; < 50 × 109/L = 2), a fibrin-related marker (D-dimer < 0.5 mg/L = 0; > 0.5 but < 4 mg/L = 2; > 4 mg/L = 3), fibrinogen (> 1.0 g/L = 0; < 1.0 g/L = 1), and PT (prolongation of PT < 3 s = 0; > 3 s but < 6 s = 1; > 6 s = 2) (3)

**Organ failure:** liver failure was defined by a TBil level of ≥12.0 mg/dL; kidney failure was defined by a serum creatinine (Cr) level of ≥ 2.0 mg/dL or the use of renal replacement therapy; cerebral failure was defined as the presence of grade III or IV HE; coagulation failure was defined by an INR >2.5; circulatory failure was defined by the use of vasoconstrictors; respiratory failure was defined by a ratio of PaO2/FiO2 ≤ 200 or SpO2 to FiO2 ≤ 214.4. (4)

**Infection:** Infection was diagnosed as follows (5): (a) spontaneous bacterial peritonitis, polymorphonuclear cell count in ascitic fluid > 250/mL; (b) pneumonia, new pulmonary infiltrate in radiologic imaging plus the presence of any respiratory symptoms (cough, sputum, dyspnoea, or pleuritic pain), any findings on auscultation (rales or crepitation), core body temperature > 38°C or  < 36°C, and WBC count > 10,000/mm^3^ or  < 4,000/mm^3^; (c) urinary tract infection, WBC count in urine > 10/high power field with positive urine culture and urinary irritation symptoms; and (d) other bacterial infections, including catheter-related infection, osteoarticular infection, skin infection, and bacteraemia of unknown cause.

**Cirrhosis:** Cirrhosis was diagnosis based on previous liver biopsy results, clinical evidence of previous decompensation and laboratory tests, endoscopy (esophageal and gastric varices) and radiological imaging of portal hypertension and/or liver nodularity. (2,6)

Pathology: (a) Presence of parenchymal nodules; (b) Differences in liver cell size and appearance; (c) Fragmentation of the biopsy specimen; (d) Fibrous septa; (e) Altered architecture and vascular relationships. Depending on the size of the nodules, there are three macroscopic types: micronodular, macronodular, and mixed cirrhosis.

Endoscopy: Esophageal and gastric varices.

Radiology: Ultrasound: The liver may appear small and nodular, with increased echogenicity and irregular-looking areas; an enlarged caudate lobe, widening of the liver fissures and enlargement of the spleen. Other radiologic tests include elastography techniques, abdominal CT and liver/bile duct MRI (MRCP).

Cirrhosis includes compensated cirrhosis and decompensated cirrhosis, the former of which is an asymptomatic compensated phase while the latter of which is a more severe pathological state marked by the development of overt clinical signs, the most frequent of which are ascites, bleeding, encephalopathy, and jaundice. (7)

**ACLF grades:** The COSSH-ACLF grades were defined according to the following criteria. ACLF grade 1 was diagnosed in: 1. patients with kidney failure only; 2. those with single liver failure with an INR ≥ 1.5 and/or kidney dysfunction (serum creatinine level ranging from 1.5 to 1.9 mg/dL) and/or HE grade I or II; 3. those with a single type of organ failure (coagulation, circulatory, or respiratory system failure) and/or kidney dysfunction and/or HE grade I or II; or 4. patients with cerebral failure only plus kidney dysfunction. ACLF grade 2 was diagnosed in patients with failure of two organ systems, while grade 3 was diagnosed in patients with failure of three or more organ systems. (2) ACLF grade-0 was defined as patients who met the inclusion criteria but have not reached the standard of ACLF grade 1. The EASL-ACLF grades were defined according to the following criteria (4). ACLF grade 1 was diagnosed in: 1. patients with kidney failure only; 2. patients with single failure of the liver, coagulation, circulation, or respiration who had a serum creatinine level ranging from 1.5 to 1.9 mg/dL and/or mild to moderate hepatic encephalopathy; 3. patients with single cerebral failure who had a serum creatinine level ranging from 1.5 to 1.9 mg/dL. EASL-ACLF grade 2 and grade 3 were diagnosed the same as those in COSSH-ACLF. EASL-ACLF grade 0 was defined as patients who met the inclusion criteria but have not reached the standard of EASL-ACLF grade 1.

**Clinical course:** To assess the clinical course of each patient, we evaluated the initial and final COSSH-ACLF grades. The initial ACLF grade was calculated at diagnosis, which occurred at admission or during hospitalization. The final grade was defined as the last available measure of ACLF grade within 28 days of diagnosis, or that before death or discharge from the hospital. Furthermore, we defined improved course as changes from ACLF-3 to ACLF-2 or -1 or -0, or from ACLF-2 to ACLF-1 or -0, or from ACLF-1 to ACLF-0 or a fixed grade of ACLF-0; steady course as no grade change; worsening course as advancing of ACLF grade, or increased number of organ failures in ACLF-3 patients. (8)

**Chronic hepatitis B:** Chronic hepatitis B is defined as hepatitis B surface antigen seropositive status beyond 6 months, with or without cirrhosis. (9)

**Prognostic scoring models:** The MELD score was calculated by the following formula: MELD = 3.78 × ln[Tbil(mg/dL)] + 11.2 × ln(INR) + 9.57 × ln[serum creatinine (mg/dL)] + 6.43; (10) The CLIF-SOFA score sums the severity grades of six organ failures, including liver, cerebra, coagulation, circulation, respiration and kidney; (4) The CLIF-C-OF score is a simplified organ function scoring system and the CLIF-C-ACLFs is a modification and optimization of CLIF-C-OFs, which is calculated as 10 × [0.33 × CLIF-OF + 0.04 × age + 0.63 × ln (white blood cell [WBC])-2]; (11) The COSSH-ACLF score is a prognostic scoring system specially designed for HBV-ACLF: COSSH-ACLF = 0.741 × INR + 0.523 × HBV-SOFA + 0.026 × age + 0.003 × Tbil. (2) COSSH-ACLF IIs is another prognostic score model for HBV-ACLF proposed by Chinese Group on the Study of Severe Hepatitis B: COSSH-ACLF IIs = 1.649 × ln (INR) + 0.457 × HE score (HE grade: 0/1, 1-2/2 and 3-4/3) + 0.425 × ln (neutrophil) (10^9^ /L) + 0.396 × ln (TB) (umol/L) + 0.576 × ln (serum urea) (mmol/L) + 0.033 × age. (12)

**Acites grade:** Grade1 ascites is mild ascites only detectable by ultrasound examination. Grade 2 ascites or moderate ascites is manifest by moderate symmetrical distension of abdomen. Grade 3 ascites is large or gross ascites with marked abdominal distension. (13)

**Methods S3: The detailed protocol of treatment for HBV-ACLF**

All patients received standard medical treatment (1,14,15), including nutrition support (glucose, vitamins, electrolytes, glutathione,etc), absolute bed rest, nucleoside analogues antiviral therapy for HBV DNA-positive patients, sodium restriction, diuretics, paracentesis combined with albumin infusion for ascites, evaluating the potential inducement and treatment with lactulose and L-ornithine aspartate for hepatic encephalopathy (HE), renal replacement for hepatorenal syndrome and uremic symptoms, prophylactic antibiotics for bacterial infection and subsequently adjusted based on the results of culture and antibiotic sensitivity tests. Patients with variceal bleeding were immediately treated with somatostatin, proton pump inhibitors, and antibiotic prophylaxis. Patients with a mean arterial pressure < 70 mmHg received fluid replacement, as well as vasoactive agents when necessary. Patients with decreased PaO2 or SpO2 received oxygen therapy, or mechanical ventilation, as necessary. (13,16,17) Artificial liver support (ALS) systems (KM8900; Kuraray, Japan), being considered as a bridge to LT or liver recovery, were frequently utilized according to the patients’ clinical state (1).

**Fig. S1 The different distribution of ACLF grades according to EASL-ACLF or COSSH-ACLF criteria in derivation cohort**


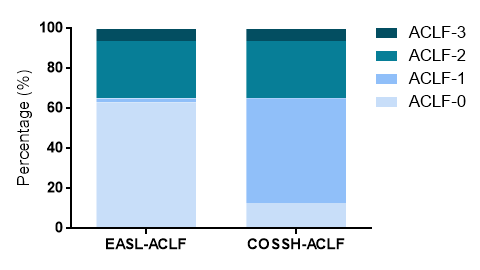


Abbreviations: ACLF: acute-on-chronic liver failure; EASL: European Association for the Study of the Liver-chronic liver failure in Cirrhosis; COSSH-ACLF: Chinese Group on the Study of Severe Hepatitis B-acute-on-chronic liver failure.

**Fig. S2 The relationship between infection and DIC score system**

**
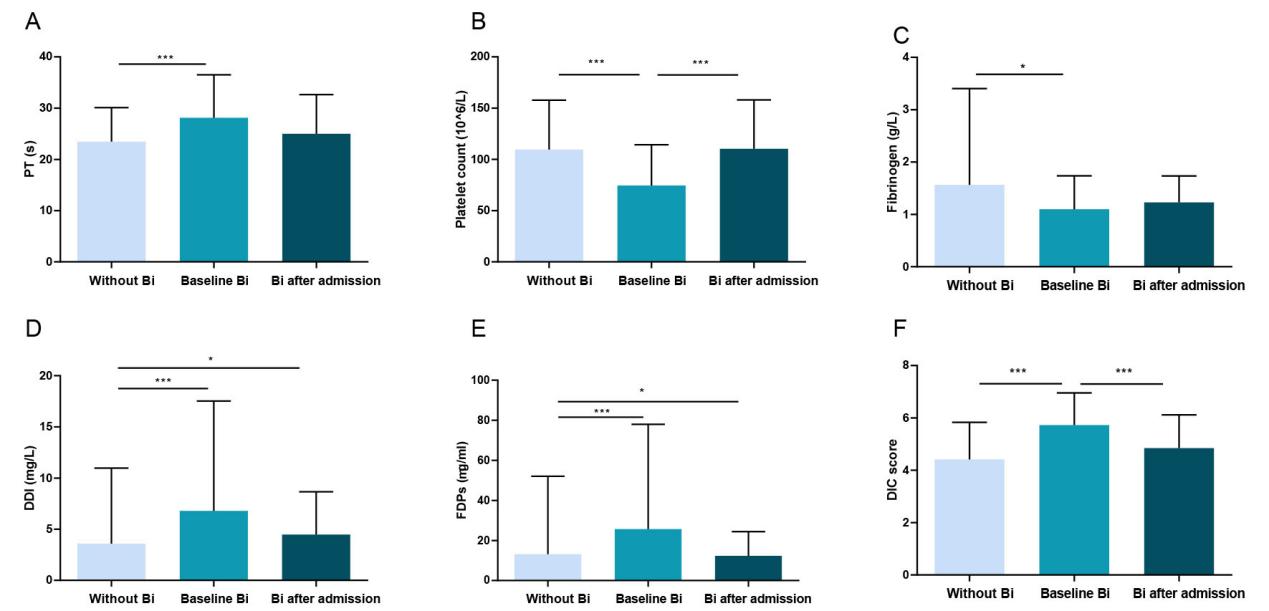
**

Association between infection and DIC score indicators (PT, Platelet count, Fibrinogen, DDI, FDPs and DIC score). Without Bi: patients who never had infection during the whole course within 90 days; Baseline Bi: patients who had infection at baseline; Bi after admission: patients without infection at baseline and developed bacterial infection within 90 days.

Platelet count was missing in one patient, and five patients lacked D-dimer and FDPs information. Some patients had been treated in other hospitals before being admitted to Huashan Hospital, so some had complications with Bi upon admission.

Abbreviations: DIC: disseminated intravascular coagulation; PT: prothrombin time; DDI: D-dimer; FDPs: fibrinogen degradation products; HBV-ACLF: hepatitis B virus-related acute-on-chronic liver failure; Bi: bacterial infection.

**Table S1** Coagulation factors associated with transplant-free 90-day mortality in patients with HBV-ACLF according to a multivariate Cox PH model.

|  | Regression coefficient | HR | 95%CI | P value |
| --- | --- | --- | --- | --- |
| INR | 0.916 | 2.499 | 1.851 - 3.374 | 0.000 |
| DIC score | 0.404 | 1.498 | 1.217 - 2.843 | 0.000 |

Abbreviations: ACLF, acute-on-chronic liver failure; HBV, hepatitis B virus; DIC, disseminated intravascular coagulation; INR, international normalized ratio.

**Table S2.** Predictors of mortality within 90-day in the univariate and multivariate Cox regression analyses in patients with HBV-ACLF

| Predictors | Univariate Cox regression | | Multivariate Cox regression | |
| --- | --- | --- | --- | --- |
|  | HR (95% CI) | P Value | HR (95% CI) | P value |
| **Age (yr)** | **1.031 (1.014-1.049)** | **0.000** | **1.031 (1.005 - 1.056)** | **0.017** |
| Underlying liver disease | - | 0.089 | - | - |
| Prior decompensation | 1.714 (1.006-2.920) | 0.048 | 1.448 (0.655-3.200) | 0.360 |
| Precipitating events | - | 0.123 | - | - |
| **Total bilirubin (μmol/L)** | **1.001 (1.001-1.001)** | **0.000** | **1.001 (1.001-1.002)** | **0.000** |
| Creatinine (μmol/L) | 1.004 (1.002-1.006) | 0.000 | 0.997 (0.992-1.001) | 0.153 |
| Sodium (mmol/L) | 0.932 (0.890 - 0.976) | 0.003 | 0.997 (0.937-1.060) | 0.912 |
| AFP (ng/ml) | 0.996 (0.993-0.999) | 0.002 | 0.999 (0.997-1.001) | 0.293 |
| White blood cell count (10^9^/L) | 1.088 (1.033-1.146) | 0.001 | 1.039 (0.966-1.117) | 0.303 |
| Hemoglobin (g/L) | 0.988 (0.979-0.998) | 0.018 | 1.013 (0.998-1.029) | 0.098 |
| Platelet count (10^9^/L) | 0.988 (0.982-0.994) | 0.000 | **-** | **-** |
| **INR** | **3.262 (2.532-4.202)** | **0.000** | **2.524 (1.628-3.914)** | **0.000** |
| Ascites grade | 1.617 (1.307-2.000) | 0.000 | 1.326 (0.965-1.822) | 0.082 |
| Bacterial infection | 3.379 (1.817-6.282) | 0.000 | 1.331 (0.611-2.899) | 0.471 |
| **Hepatic encephalopathy grade** | **2.170 (1.823-2.584)** | **0.000** | **1.494 (1.117-1.999)** | **0.007** |
| **DIC score** | **1.876 (1.570- 2.241)** | **0.000** | **1.397 (1.040-1.875)** | **0.026** |
| Artificial liver | 1.895 (1.181-3.042) | 0.008 | 1.504 (0.806-2.809） | 0.200 |

Abbreviations: HBV-ACLF, hepatitis B virus-related acute-on-chronic liver failure; INR, international normalized ratio; OR, odds ratio; CI, confidence interval; DIC, disseminated intravascular coagulation; Alpha fetoprotein, AFP.

Bold-face font represents factors that are significant predictors of prognosis in multivariate analyses.

The multivariate Cox regression model was fitted with enter method using clinically and statistically baseline factors that had been screened in univariate analysis.

**Table S3.** Comparison of the predictive value of prognostic scoring systems for patients with HBV-ACLF in derivation cohort

|  | 28-day | | 90-day | |
| --- | --- | --- | --- | --- |
|  | auROC | P value | auROC | P value |
| CLIF-C OF-DICs | 0.924 |  | 0.936 |  |
| COSSH-ACLFs | 0.904 | 0.4339 | 0.903 | 0.2545 |
| COSSH-ACLF-IIs | 0.905 | 0.4439 | 0.901 | 0.1990 |
| CLIF-C OFs | 0.900 | 0.5247 | 0.870 | 0.0210 |
| CLIF-SOFA | 0.895 | 0.4337 | 0.863 | 0.0192 |
| CLIF-C ACLFs | 0.888 | 0.0505 | 0.879 | 0.0164 |
| MELD | 0.836 | 0.0033 | 0.830 | 0.0007 |

The P values represents the significance of difference between the auROC of CLIF C-OF-DICs and six other prognostic scoring models.

Abbreviations: auROC, area under the receiver operating characteristic curve; ACLF, acute-on-chronic liver failure; DIC, disseminated intravascular coagulation. HBV, hepatitis B virus; CLIF-SOFA, chronic liver failure-sequential organ failure assessment; CLIF-C OF-DICs: a novel prognostic score based on age, DIC score, and CLIF-C OFs; COSSH-ACLFs, Chinese Group on the Study of Severe Hepatitis B-ACLF score; CLIF-C OFs, CLIF Consortium Organ Function score; COSSH-ACLF IIs: Chinese Group on the Study of Severe Hepatitis B-ACLF II score; CLIF-C ACLFs, CLIF Consortium ACLF score; MELD, Model for End-stage Liver Disease;

**Table S4.** Clinical and laboratory characters at baseline between derivation cohort and external validation cohort.

|  | **Deviation (n = 163)** | **Validation (n = 82)** | P value |
| --- | --- | --- | --- |
| **Clinical data** |  |  |  |
| Age (yr) | 46 (37-56) | 48 (40-53) | 0.851 |
| Male sex, % (no.) | 91.4 (149) | 86.6 (71) | 0.239 |
| Underlying liver disease, % (no.) |  |  | 0.031 |
| Chronic hepatitis B | 49.1 (80) | 43.9 (36) |  |
| Compensated cirrhosis | 31.3 (51) | 46.3 (38) |  |
| Decompensated cirrhosis | 19.6 (32) | 9.8 (8) |  |
| Precipitating events |  |  |  |
| HBV reactivation, % (no.) | 47.9 (78) | 54.9 (45) | 0.299 |
| Bacterial infection, % (no.) | 5.5 (9) | 7.3 (6) | 0.580 |
| Complications, % (no.) |  |  |  |
| Ascites | 69.3 (113) | 68.3 (56) | 0.869 |
| GI hemorrhage | 7.4 (12) | 3.7 (3) | 0.254 |
| Bacterial infection | 63.8 (104) | 58.5 (48) | 0.423 |
| Artificial liver, % (no.) | 28.2 (46) | 19.5 (16) | 0.139 |
| **Laboratory data** |  |  |  |
| Alanine aminotransferase (U/L) | 157 (72-431) | 113 (67-153) | 0.185 |
| Albumin (g/L) | 32 (29-36) | 32 (29-35) | 0.419 |
| Total bilirubin (μmol/L) | 307.1 (72.0-431.0) | 388.45 (241.7-473.5) | 0.011 |
| Creatinine (μmol/L) | 69 (58-86) | 62 (52-72) | 0.000 |
| White blood cell count (10^9^/L) | 6.76 (5.04-10.36) | 5.93 (4.79-8.48) | 0.155 |
| Hemoglobin (g/L) | 120 (104-136) | 116 (104-135) | 0.762 |
| Platelet count (10^9^/L) | 90 (62-121) | 85 (60-122) | 0.881 |
| INR | 2.11 (1.80-2.64) | 2.27 (1.90-2.77) | 0.147 |
| DIC score | 5 (4-6) | 4 (4-5) | 0.710 |
| FIB (g/L) | 1.17 (0.8-1.5) | 1.41 (1.07-1.91) | 0.001 |
| DDI (μg/ml) | 3.05 (1.42-4.92) | 2.13 (1.34-3.17) | 0.033 |
| FDPs (μg/ml) | 7.5 (3.6 -14.6) | 5.9 (3.5-12.4) | 0.191 |
| **ACLF grade, % (no.)** |  |  | 0.851 |
| 0 | 12.9 (21) | 17.1 (14) |  |
| 1 | 52.1 (85) | 51.2 (42) |  |
| 2 | 28.8 (47) | 29.3 (24) |  |
| 3 | 6.1 (10) | 4.9 (4) |  |
| **28-day mortality** | 35.0 (57) | 19.5 (16) | 0.0087 |
| **90-day mortality** | 44.8 (73) | 42.7 (35) | 0.0746 |

Abbreviations: HBV, hepatitis B virus; INR, international normalized ratio; COSSH-ACLF, Chinese Group on the Study of Severe Hepatitis B-ACLF; GI, gastrointestinal; DIC, disseminated intravascular coagulation; FIB, fibrinogen; FDPs, fibrinogen degradation products; DDI, D-dimer.

Data are expressed as the median (interquartile range) or percent (number).

**Table S5.** Comparison of the predictive value of prognostic scoring systems for patients with HBV-ACLF in external validation cohort.

|  | 28-day | | 90-day | |
| --- | --- | --- | --- | --- |
|  | auROC | P value | auROC | P value |
| CLIF-C OF-DICs | 0.791 |  | 0.812 |  |
| COSSH-ACLFs | 0.804 | 0.8535 | 0.797 | 0.8099 |
| COSSH-ACLF IIs | 0.845 | 0.1117 | 0.790 | 0.9767 |
| CLIF-C ACLFs | 0.784 | 0.8724 | 0.737 | 0.0787 |
| CLIF-SOFA | 0.744 | 0.3914 | 0.741 | 0.1885 |
| CLIF-C OFs | 0.744 | 0.1424 | 0.767 | 0.1817 |
| MELD | 0.804 | 0.8333 | 0.774 | 0.4903 |

The P values represents the significance of difference between the auROC of CLIF C-OF-DICs and six other prognostic scoring models.

Abbreviations: auROC, area under the receiver operating characteristic curve; ACLF, acute-on-chronic liver failure; DIC, disseminated intravascular coagulation. HBV, hepatitis B virus; CLIF-SOFA, chronic liver failure-sequential organ failure assessment; CLIF-C OF-DICs: a novel prognostic score based on age, DIC score, and CLIF-C OFs; COSSH-ACLFs, Chinese Group on the Study of Severe Hepatitis B-ACLF score; CLIF-C OFs, CLIF Consortium Organ Function score; COSSH-ACLF IIs: Chinese Group on the Study of Severe Hepatitis B-ACLF II score; CLIF-C ACLFs, CLIF Consortium ACLF score; MELD, Model for End-stage Liver Disease;

**References**

1. Sarin SK, Choudhury A, Sharma MK, Maiwall R, Al Mahtab M, Rahman S, et al. Acute-on-chronic liver failure: consensus recommendations of the Asian Pacific association for the study of the liver (APASL): an update. *Hepatol Int.* (2019) 13:353-390. doi: 10.1007/s12072-019-09946-3
2. Wu T, Li J, Shao L, Xin J, Jiang L, Zhou Q, et al. Development of diagnostic criteria and a prognostic score for hepatitis B virus-related acute-on-chronic liver failure. *Gut.* (2018) 67:2181-2191. doi: 10.1136/gutjnl-2017-314641
3. Taylor FB, Jr., Toh CH, Hoots WK, Wada H, Levi M. Towards definition, clinical and laboratory criteria, and a scoring system for disseminated intravascular coagulation. *Thromb Haemost.* (2001) 86:1327-1330.
4. Moreau R, Jalan R, Gines P, Pavesi M, Angeli P, Cordoba J, et al. Acute-on-chronic liver failure is a distinct syndrome that develops in patients with acute decompensation of cirrhosis. *Gastroenterology.* (2013) 144:1426-1437, 1437.e1421-1429. doi: 10.1053/j.gastro.2013.02.042
5. Nahon P, Lescat M, Layese R, Bourcier V, Talmat N, Allam S, et al. Bacterial infection in compensated viral cirrhosis impairs 5-year survival (ANRS CO12 CirVir prospective cohort). *Gut.* (2017) 66:330-341. doi: 10.1136/gutjnl-2015-310275
6. Foucher J, Chanteloup E, Vergniol J, Castéra L, Le Bail B, Adhoute X, et al. Diagnosis of cirrhosis by transient elastography (FibroScan): a prospective study. *Gut.* (2006) 55:403-408. doi: 10.1136/gut.2005.069153
7. EASL Clinical Practice Guidelines for the management of patients with decompensated cirrhosis. *J Hepatol.* (2018) 69:406-460. doi: 10.1016/j.jhep.2019.01.037
8. Gustot T, Fernandez J, Garcia E, Morando F, Caraceni P, Alessandria C, et al. Clinical Course of acute-on-chronic liver failure syndrome and effects on prognosis. *Hepatology.* (2015) 62:243-252. doi: 10.1002/hep.27849
9. Sarin SK, Kumar M, Lau GK, Abbas Z, Chan HL, Chen CJ, et al. Asian-Pacific clinical practice guidelines on the management of hepatitis B: a 2015 update. *Hepatol Int.* (2016) 10:1-98. doi: 10.1007/s12072-015-9675-4
10. Kamath PS, Kim WR. The model for end-stage liver disease (MELD). *Hepatology.* (2007) 45:797-805. doi: 10.1002/hep.21563
11. Jalan R, Saliba F, Pavesi M, Amoros A, Moreau R, Ginès P, et al. Development and validation of a prognostic score to predict mortality in patients with acute-on-chronic liver failure. *J Hepatol.* (2014) 61:1038-1047. doi: 10.1016/j.jhep.2014.06.012
12. Li J, Liang X, You S, Feng T, Zhou X, Zhu B, et al. Development and validation of a new prognostic score for hepatitis B virus-related acute-on-chronic liver failure. *J Hepatol.* 2021. doi: 10.1016/j.jhep.2021.05.026
13. Moore KP, Wong F, Gines P, Bernardi M, Ochs A, Salerno F, et al. The management of ascites in cirrhosis: report on the consensus conference of the International Ascites Club. *Hepatology.* (2003) 38:258-266. doi: 10.1053/jhep.2003.50315
14. Vilstrup H, Amodio P, Bajaj J, Cordoba J, Ferenci P, Mullen KD, et al. Hepatic encephalopathy in chronic liver disease: 2014 Practice Guideline by the American Association for the Study of Liver Diseases and the European Association for the Study of the Liver. *Hepatology.* (2014) 60:715-735. doi: 10.1002/hep.27210
15. EASL clinical practice guidelines on the management of ascites, spontaneous bacterial peritonitis, and hepatorenal syndrome in cirrhosis. *J Hepatol.* (2010) 53:397-417. doi: 10.1016/j.jhep.2010.05.004
16. Arroyo V, Jiménez W. Complications of cirrhosis. II. Renal and circulatory dysfunction. Lights and shadows in an important clinical problem. *J Hepatol.* (2000) 32:157-170. doi: 10.1016/s0168-8278(00)80423-7
17. Garcia-Tsao G, Bosch J. Management of varices and variceal hemorrhage in cirrhosis. *N Engl J Med.* (2010) 362:823-832. doi: 10.1056/NEJMra0901512
